# A positive feedback loop between miR‐181b and STAT3 that affects Warburg effect in colon cancer via regulating PIAS3 expression

**DOI:** 10.1111/jcmm.13786

**Published:** 2018-07-28

**Authors:** Xiaolin Pan, Jin Feng, Zhenhua Zhu, Linhua Yao, Shijie Ma, Bo Hao, Guoxin Zhang

**Affiliations:** ^1^ Department of Gastroenterology The First Affiliated Hospital of Nanchang University Nanchang China; ^2^ Department of General Surgery The Third Affiliated Hospital of Soochow University Changzhou China; ^3^ Department of Gastroenterology The First Affiliated Hospital of Huzhou Teachers College Huzhou China; ^4^ Department of Gastroenterology The First Affiliated Hospital of Nanjing Medical University Nanjing China

**Keywords:** MicroRNA‐181b, PIAS3, positive feedback loop, STAT3, Warburg effect

## Abstract

This study aimed to investigate the relationship between the expression of microRNA (miR)‐181b, protein inhibitor of activated STAT3 (PIAS3) and STAT3, and to examine the function of the miR‐181b/PIAS3/STAT3 axis on the Warburg effect and xenograft tumour growth of colon cancer. Moreover, a positive feedback loop between miR‐181b and STAT3 that regulated the Warburg effect in colon cancer was explored. A luciferase reporter assay was used to identify whether PIAS3 was a direct target of miR‐181b. The gain‐of‐function and loss‐of‐function experiments were performed on HCT 116 cells to investigate the effect of miR‐181b/PIAS3/STAT3 on the Warburg effect and xenograft tumour growth of colon cancer, as determined by commercial kits and xenograft experiments. The relationship between the expression of miR‐181b, PIAS3 and STAT3 in HCT 116 and HT‐29 cells was determined using RT‐qPCR and Western blot. We found miR‐181b was a direct regulator of PIAS3. miR‐181b promoted the Warburg effect and the growth of colon cancer xenografts; however, these effects could be reversed by PIAS3. miR‐181b expression interacted with STAT3 phosphorylation in a positive feedback loop in colon cancer cells via regulating PIAS3 expression. In conclusion, this study for the first time demonstrated that miR‐181b contributed to the Warburg effect and xenograft tumour growth of colon cancer by targeting PIAS3. Moreover, a positive feedback loop between miR‐181b and STAT3 that regulated the Warburg effect in colon cancer was also demonstrated. This study suggested miR‐181b/PIAS3/STAT3 axis as a novel target for colon cancer treatment.

## 1 INTRODUCTION

Colon cancer is a leading cause of cancer death in the worldwide.^1^, ^2^ About 1 million people suffer colon cancer every year, and it causes 0.6 million deaths annually around the world.^3^ Despite advances in surgical treatment, the 5‐year overall survival rate of patients with metastatic colon cancer is only 5%‐10%.^4^ Therefore, it is necessary to investigate the molecular mechanisms underlying colon cancer development so as to contrive novel strategies for colon cancer treatment.

Cancer cells rewire their metabolism to promote cancer progression. The common feature of this altered metabolism is that cancer cells preferentially produce energy by a high rate of glycolysis followed by lactic acid fermentation in the cytosol,^5^, ^6^ rather than by a comparatively low rate of glycolysis followed by oxidation of pyruvate in mitochondria as in most normal cells.^7^ This phenomenon is referred as the Warburg effect, which has been documented for over 90 years. The Warburg effect is considered as one of the most fundamental metabolic alteration associated with malignant transformation; however, it remains unclear how the Warburg effect is regulated during tumour progression.

MicroRNAs (miRNAs or miR) are a class of small non‐coding RNAs that regulate gene expression at the post‐transcriptional and translational levels through binding to the 3′untranslated region (3′UTR) of target mRNAs. The roles of miRNAs in cancer progression have been well‐documented and several miRNAs such as miR‐223, miR‐133, and miR‐200 family have been implicated in the regulation of aerobic glycolysis in cancer.^8^ MiR‐181b is an important miRNA which is correlated with tumorigenesis.^9^ MiR‐181b has been shown to exert its effect on cancer by regulating cell proliferation, apoptosis, invasion and migration.^10^, ^11^ Recently, the role of miR‐181b in regulating aerobic glycolysis in cancer has attracted much concern.^12^, ^13^

Signal transducer and activator of transcription 3 (STAT3) is a transcription factor that mediates cellular responses to various cytokines and growth factors. Recently, some studies reveal that STAT3 acts as a master regulator of aerobic glycolysis.^14^, ^15^, ^16^ STAT3 regulates the Warburg effect via promoting aerobic glycolysis and downregulating mitochondrial activity.^16^ Protein inhibitor of activated STAT 3 (PIAS3) is a specific inhibitor of STAT3. It has been shown that ectopic expression of PIAS3 in cancer cells can suppress the transcriptional activity of STAT3 and inhibit tumour growth.^17^, ^18^

In the present study, we explored the relationship between miR‐181b (miR‐181b‐5p), PIAS3 and STAT3, and investigated the function of miR‐181b/PIAS3/STAT3 axis on the Warburg effect and xenograft tumour growth of colon cancer. Moreover, a miR‐181b‐STAT3 positive feedback loop that contributed to the Warburg effect in colon cancer cells was demonstrated as well.

## 2 MATERIALS AND METHODS

### 2.1 Cell culture and transfection

HCT 116, HT‐29, and HEK‐293T cells were purchased from the Cell Bank of the Chinese Academy of Sciences (Shanghai, China). Cells were cultured in RPMI 1640 medium (Gibco Life Technologies, Carlsbad, CA) containing 10% foetal bovine serum (FBS; Gibco Life Technologies) and maintained at 37°C in a humidified atmosphere with 5% CO_2_. The miR‐181b mimic (miR‐181b)/inhibitor (as‐miR‐181b), miR‐181b negative control (miR NC), as‐miR NC, STAT3 siRNAs (siRs), PIAS3 siRs, scramble (Scr) siR, and pFlag‐PIAS3 plasmid were designed and synthesized by Gene Pharma (Shanghai, China). The sequences of miRNAs and siRs were shown in Table 1. Cell transfection (plasmid 4.0 μg, RNA 100 pmol/L) was performed using Lipofectamine 2000 (Invitrogen, Carlsbad, CA) according to the manufacturer's instructions.

**Table 1:** The sequences of miRNAs and siRNAs

miRNAs or siRNAs	Sequences
miR‐181b	5′‐AACAUUCAUUGCUGUCGGUGGGU‐3′
	5′‐CCACCGACAGCAAUGAAUGUUUU‐3′
miR NC	5′‐UUCUCCGAACGUGUCACGUTT‐3′
	5′‐ACGUGACACGUUCGGAGAATT‐3′
as‐miR‐181b	5′‐ACCCACCGACAGCAAUGAAUGUU‐3′
as‐miR NC	5′‐CAGUACUUUUGUGUAGUACAA‐3′
STAT3 siR‐1	5′‐CCCGGAAAUUUAACAUUCUTT‐3′
	5′‐AGAAUGUUAAAUUUCCGGGTT‐3′
STAT3 siR‐2	5′‐GGGACCUGGUGUGAAUUAUTT‐3′
	5′‐AUAAUUCACACCAGGUCCCTT‐3′
STAT3 siR‐3	5′‐GGUACAUCAUGGGCUUUAUTT‐3′
	5′‐AUAAAGCCCAUGAUGUACCTT‐3′
PIAS3 siR‐1	5′‐CAUCCAAGGUUUAGAUUUATT‐3′
	5′‐UAAAUCUAACCUUGGAUUGTT‐3′
PIAS3 siR‐2	5′‐CUACAAAAACUCAGAGCAATT‐3′
	5′‐UUGCUCUGAGUUUUUGUAGTT‐3′
PIAS3 siR‐3	5′‐CAAACAGACAGGUGGAAAATT‐3′
	5′‐UUUUCCACCUGUCUGUUUGTT‐3′
Scr siR	5′‐UUCUCCGAACGUGUCACGUTT‐3′
	5′‐ACGUGACACGUUCGGAGAATT‐3′

### 2.2 Xenograft experiments

This study was approved by the Ethics Committee of Nanjing Medical University. The BALB/C nude mice (female, 4 weeks, 18‐22 g) were purchased from the Laboratory Animal Center of Nanjing Medical University. The mice were randomly distributed to 6 groups: as‐miR negative control (NC) group, as‐miR‐181b group, pFlag group, pFlag‐PIAS3 group, as‐miR‐181b + Scramble (Scr) siR group, and as‐miR‐181b + PIAS3 siR group. The protocol referred to a previous publication.^19^ In brief, HCT 116 cells (5 × 10^6^) transfected with as‐miR/siR/pFlag were injected subcutaneously into the right back of nude mice. Ten days after inoculation, the mice were intraperitoneally injected with 0.5 mL of as‐miR NC (100 nmol/L), as‐miR‐181b (100 nmol/L),pFlag (100 nmol/L), pFlag‐PIAS3(100 nmol/L), as‐miR‐181b + Scr siR (100 nmol/L)),or as‐miR‐181b + PIAS3 siR(100 nmol/L)every 5 days. Tumour growth was monitored every 5 days for 5 weeks, and tumour volume was calculated using the formula *V* (mm^3^) = *a* × *b* ^2^/2, where *a* was the largest diameter and *b* was the perpendicular diameter.^20^

### 2.3 The assay of glucose uptake and lactic acid production

To determine glucose uptake and lactic acid production, after transfection for 24 hours, culture medium was collected and assayed using the Glucose Assay Kit and Lactate Assay Kit (Biovison, Milpitas, California, USA) following the manufacturer's instructions.

### 2.4 Luciferase reporter assay

HEK‐293T cells were plated at 5 × 10^4^ cells per well in a 24‐well plate. pGL‐PIAS3 3′UTR wild‐type (wt) and pGL‐PIAS3 3′UTR mutant (mut) plasmids were synthesized by Invitrogen. The plasmids were cotransfected with the miR‐181b or miR‐NC into the cells using Lipofectamine 2000. pRL‐TK *Renilla* luciferase plasmid was used as the internal control. Following transfection for 24 hours, luciferase activity was measured using a Dual‐Luciferase Reporter Assay System (Promega, Madison, WI) with a Glomax Detector (Promega).

### 2.5 Reverse transcription‐quantitative polymerase chain reaction (RT‐qPCR)

After transfection for 48 hours, total RNA was isolated from cells using the TRIzol Reagent (Invitrogen) according to the manufacturer's instructions. 1 μg of total RNA was transcribed to cDNA using the M‐MLV Reverse Transcriptase (Invitrogen). RT‐qPCR was performed using SYBR Premix Ex Taq Real‐Time kit (Takara, Japan) on a StepOne Real‐Time PCR System (Applied Biosystems, Foster City, CA). The primers used were: miR‐181b, 5′‐GCGGATCATTCATTGCTGTCG‐3′(forward), 5′‐GTGCAGGGTCCGAGGT‐3′(reverse); PIAS3, 5′‐GCCGACATGGACGTGTCCTGTG‐3′(forward), 5′‐TTCCCTCCTGGACTGCGCTGTAC‐3′(reverse); β‐actin, 5′‐AGCGAGCATCCCCCAAAGTT‐3′(forward), 5′‐GGGCACGAAGGCTCATCATT‐3′(reverse); U6, 5′‐CTCGCTTCGGCAGCACATA‐3′(forward), 5′‐GTGCAGGGTCCGAGGT‐3′(reverse). β‐actin and U6 were used as the control.

### 2.6 Western blot

After transfection for 24 hours, cells were lyzed in lysis buffer (Beyotime, Shanghai, China) with the protease inhibitor (Roche, Mannheim, Germany). The protein concentration was examined with a BCA Protein Assay kit (Pierce, Rockford, IL). For sodium dodecyl sulphate polyacrylamide (SDS) gel electrophoresis, equal quantities of total protein (30 μg) were separated on 10% SDS‐PAGE and then transferred onto polyvinylidene fluoride membranes (Millipore, Billerica, MA). After blocking in nonfat milk at room temperature for 1 hour, the membranes were incubated with the specific antibodies against PIAS3 (Abcam, Cambridge, MA), STAT3 (Cell Signalling Technology, Beverly, MA), p‐STAT3 (Tyr705) (Cell Signalling Technology) and β‐actin (Cell Signalling Technology) at 4°C overnight. After washing with Tris‐buffered saline‐tween (TBS‐T), the membranes were then incubated with horseradish peroxidase (HRP)‐conjugated secondary antibody at room temperature for 1‐2 hours. Protein bands were visualized using the SuperSignal West Pico Chemiluminescent Substrate (Pierce). The Western blots were quantified using Gel‐Pro analyzer software (v4.5, Media Cybernetics, Rockville, Maryland, USA).

### 2.7 Statistical analysis

Statistical analysis was performed using one‐way analysis of variance (ANOVA) followed by Tukey's post hoc test with SPSS software (version 17.0; Chicago, IL). The data were obtained from at least three independent experiments and presented as the mean ± standard deviation (SD). *P* < 0.05 was considered to indicate a statistically significant result.

## 3 RESULTS

### 3.1 miR‐181b directly targets PIAS3

**Figure 1:**
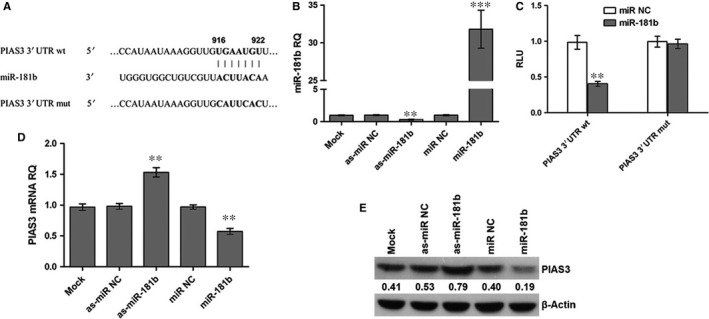
miR‐181b Directly Targets PIAS3. A, The putative transcription binding sites of miR‐181b and PIAS3 3′UTR. B, Relative mRNA expression of miR‐181b in HCT 116 cells following transfection with the miR‐181b mimic or inhibitor. **P < 0.01 and ***P < 0.001. C, The relative luciferase activity of wild‐type or mutant PIAS3 3′UTR in HEK293 cells following transfection with the miR NC or miR‐181b. **P < 0.01. D, Relative mRNA expression of PIAS3 in HCT 116 cells following transfection with the miR‐181b mimic or inhibitor. **P < 0.01. E, Relative protein expression of PIAS3 in HCT 116 cells following transfection with the miR‐181b mimic or inhibitor. RQ: relative quantity; Mock: transfection reagent only

We used Targetscan, Pictar, and miRanda to search for the putative transcription binding sites of miR‐181b and PIAS3 3′UTR, and the predicted sites were shown in Figure 1A. miR‐181b mimic or inhibitor was transfected into the cells to overexpress or knockdown miR‐181b expression, respectively. As demonstrated in Figure 1B, miR‐181b expression was significantly up‐regulated in mimic‐transfected cells, but decreased in inhibitor‐transfected cells. To examine whether PIAS3 was a direct target of miR‐181b, pGL‐PIAS3 3′UTR wt or pGL‐PIAS3 3′UTR mut was cotransfected with the miR‐181b mimic or miR NC. The results from luciferase reporter assay revealed that compared with the cells transfected with the miR NC, the luciferase activity of pGL‐PIAS3 3′UTR wt was significantly suppressed in the cells transfected with the miR‐181b mimic; however, there was no significant difference in the luciferase activity of pGL‐PIAS3 3′UTR mut between miR NC and miR‐181b mimic ‐transfected cells (Figure 1C). Subsequently, we examined the effect of miR‐181b on PIAS3 mRNA and protein expression. The results from RT‐PCR and Western blot showed that the relative mRNA and protein levels of PIAS3 were significantly decreased in the miR‐181b mimic‐transfected cells, but increased in the miR‐181b inhibitor‐transfected cells compared with the miR NC‐transfected cells (Figures 1D,E and S1).

### 3.2 PIAS3 reverses the effect of miR‐181b on the Warburg effect in colon cancer cells

**Figure 2:**
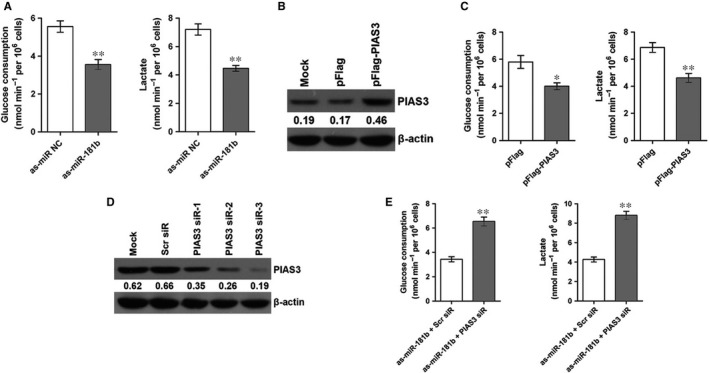
PIAS3 Reverses the Effect of miR‐181b on the Warburg Effect in Colon Cancer Cells. A, miR‐181b knockdown inhibits the glucose consumption and lactic acid production. **P < 0.01. B, Relative protein expression of PIAS3 in HCT 116 cells following transfection with the pFlag‐PIAS3 or pFlag. C, PIAS3 overexpression inhibits the glucose consumption and lactic acid production. *P < 0.05 and **P < 0.01. D, Relative protein expression of PIAS3 in HCT 116 cells following transfection with the PIAS3 siRs. E, PIAS3 reverses the effect of miR‐181b on glucose consumption and lactic acid production. **P < 0.01. Mock: transfection reagent only

To reveal the effect of miR‐181b on aerobic glycolysis in colon cancer cells, miR‐181b inhibitor was transfected into the HCT 116 cells to knockdown miR‐181b, and then glucose and lactic acid were detected using the commercial kits. As shown in Figure 2A, knockdown of miR‐181b significantly decreased the glucose consumption and lactic acid production. pFlag‐PIAS3 plasmid was transfected into the HCT 116 cells to overexpress PIAS3, and the results from western blot demonstrated that compared with the control cells, the relative protein level of PIAS3 was obviously increased in the cells following transfection with the pFlag‐PIAS3 plasmid (Figure 2B). Further, we found the glucose consumption and lactic acid production were significantly inhibited in the PIAS3‐overexpressing cells (Figure 2C).

To investigate whether PIAS3 mediated the effect of miR‐181b on aerobic glycolysis in colon cancer cells, we synthesized three PIAS3 siRs to knockdown PIAS3 and found that PIAS3 siR‐3 was the most efficient siRNA (Figure 2D). Subsequently, PIAS3 siR‐3/Scr siRNA were cotransfected with as‐miR‐181b into the HCT 116 cells, and the glucose consumption and lactic acid production were determined. As shown in Figure 2E, compared with the control, HCT 116 cells with suppressed expression of PIAS3 showed significantly increased glucose consumption and lactic acid production.

### 3.3 PIAS3 reverses the effect of miR‐181b on xenograft tumour growth

**Figure 3:**
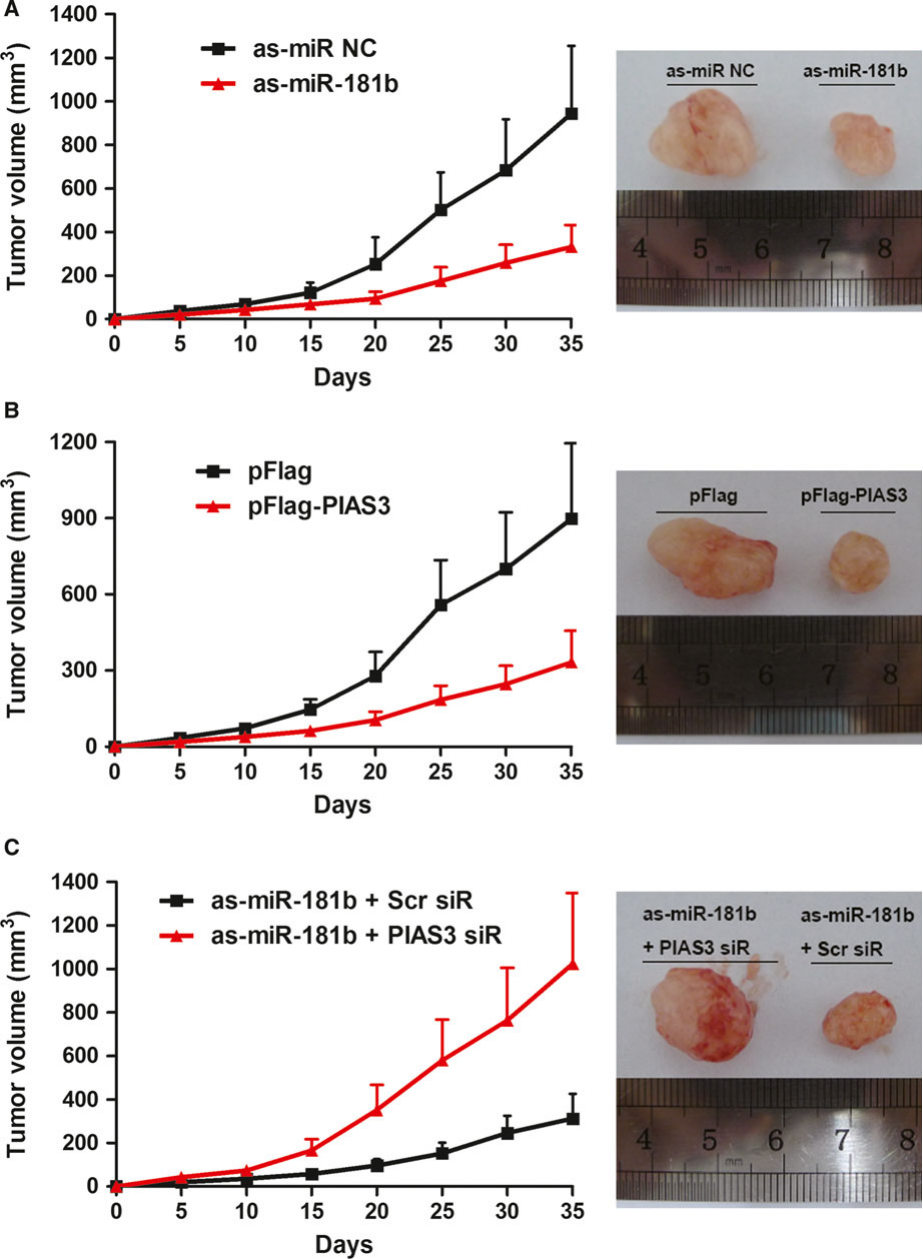
siPIAS3 Reverses the Effect of miR‐181b on Xenograft Tumour Growth. A, miR‐181b knockdown inhibits xenograft tumour growth. B, PIAS3 overexpression inhibits xenograft tumour growth. C, siPIAS3 reverses the effect of miR‐181b on xenograft tumour growth

To investigate the effect of miR‐181b on xenograft tumour growth, HCT 116 cells transfected with as‐miR‐181b or as‐miR NC were injected into the nude mice, and the tumour growth was monitored and measured. It was observed that the tumour volume was significantly decreased in miR‐181b‐knockdown tumours compared with that of the control tumours (Figure 3A).As shown in Figure 3B, the tumour volumes of xenograft tumours with a high level of PIAS3 expression were decreased compared with that of the control tumours. Furthermore, we found the tumour volume in the as‐miR‐181b + PIAS3 siR group was larger than that in the as‐miR‐181b + Scr siR group (Figure 3C).

### 3.4 PIAS3 reverses the effect of miR‐181b on the phosphorylation of STAT3

**Figure 4:**
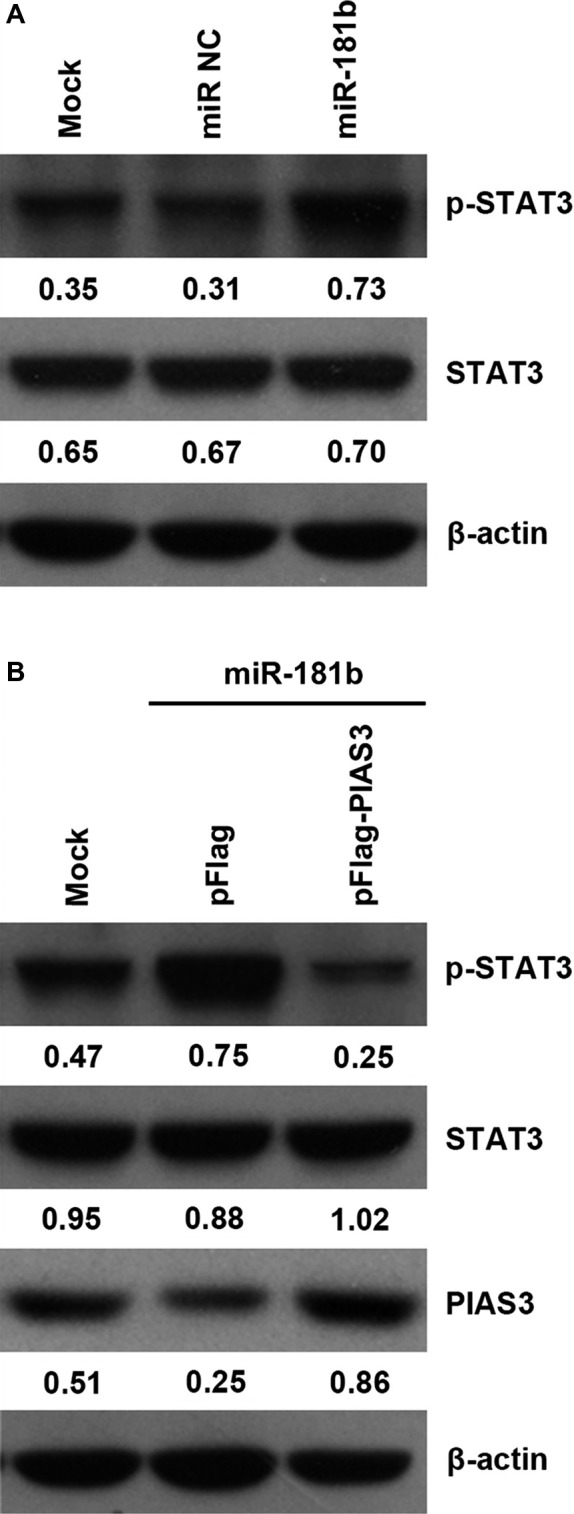
PIAS3 Reverses the Effect of miR‐181b on the Phosphorylation of STAT3. A, Transfection with the miR‐181b obviously increased the relative protein level of p‐STAT3. B, PIAS3 reverses the effect of miR‐181b on the phosphorylation of STAT3. Mock: transfection reagent only

The miR‐181b and miR NC were transfected into the HCT 116 cells. The as‐miR‐181b and as‐miR NC were transfected into the HT‐29 cells. Western blot was performed to examine the protein expression of STAT3 and p‐STAT3. As shown in Figure 4A, transfection with the miR‐181b significantly increased the relative protein level of p‐STAT3. Figure S1 showed that transfection with the as‐miR‐181b significantly decreased the protein level of p‐STAT3. However, the promotive effect of miR‐181b on the phosphorylation of STAT3 was reversed by transfection with pFlag‐PIAS3 (Figure 4B).

### 3.5 PIAS3 overexpression suppresses p‐STAT3 and miR‐181b expression

**Figure 5:**
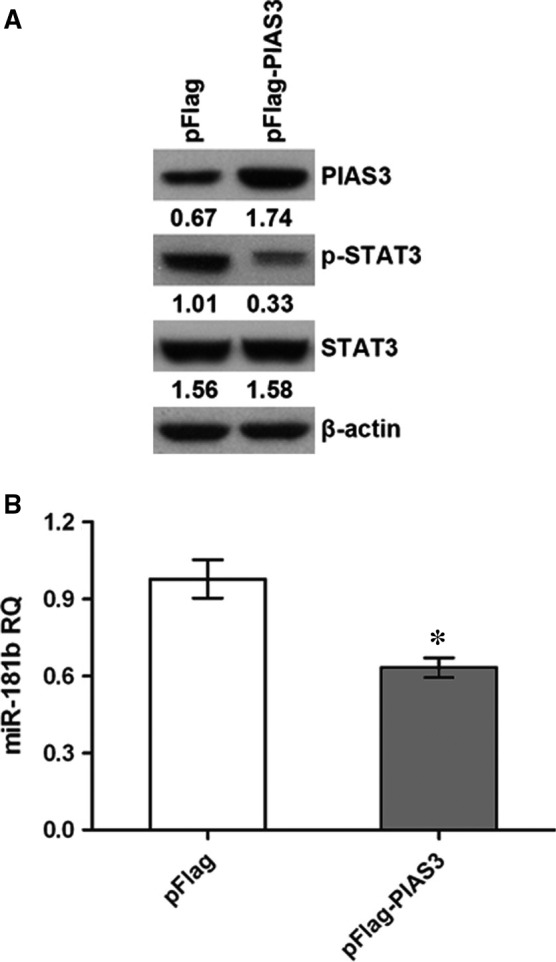
Effect of PIAS3 on p‐STAT3 and miR‐181b Expression in HCT 116 Cells. A, PIAS3 overexpression suppresses p‐STAT3 expression. B, PIAS3 overexpression suppresses miR‐181b expression. *P < 0.05. RQ: relative quantity

The pFlag‐PIAS3 was transfected into the HCT 116 and HT‐29 cells to overexpress PIAS3. Subsequently, the protein expression of STAT3 and p‐STAT3 was examined by western blot, and the miR‐181b expression was detected by RT‐qPCR. We found PIAS3 overexpression could suppress the p‐STAT3 and miR‐181b expression (Figures 5 and S2).

### 3.6 STAT3 suppression down‐regulates miR‐181b and up‐regulates PIAS3

**Figure 6:**
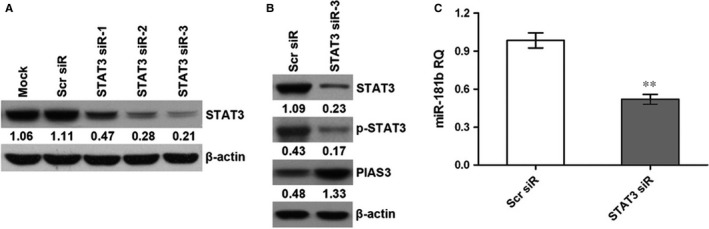
Effect of STAT3 on miR‐181b and PIAS3 Expression in HCT 116 Cells. A, STAT3 siRs downregulate STAT3. B, STAT3 suppression upregulates PIAS3. C, STAT3 suppression downregulates miR‐181b. **P < 0.01. RQ: relative quantity; Mock: transfection reagent only

To knockdown STAT3 expression in HCT 116 cells, three STAT3 siRs were transfected into the cells, and the results of western blot showed that the relative protein level of STAT3 was obviously decreased in STAT3 siR‐transfected cells (Figure 6A). Among these siRs, STAT3‐siR3 was the most efficient. Therefore, we selected STAT3‐siR3 for subsequent experiments. We found the expression of STAT3 and p‐STAT3 was obviously decreased in the HCT 116 and HT‐29 cells following STAT3‐siR3 transfection (Figures 6B and S3A). As shown in Figures 6B,C and S3, STAT3 suppression resulted in an increase in the PIAS3 protein level and a decrease in the miR‐181b level.

## 4 DISCUSSION

In this study, we used bioinformatics softwares (Targetscan, http://www.targetscan.org; Pictar, http://pictar.mdc-berlin.de/; and miRanda, http://www.microrna.org/microrna/home.do) to identify the potential targets of miR‐181b, and found PIAS3 was a predicted one. Through luciferase reporter assay, we found miR‐181b mimic was able to suppress luciferase activity encoded by a reporter gene fused to the PIAS3 3′UTR. In addition, the mRNA and protein levels of PIAS3 were down‐regulated by miR‐181b mimic, and up‐regulated by miR‐181b inhibitor. These results for the first time validated miR‐181b as a direct regulator of PIAS3.

Previous studies had revealed that miR‐181b expression was altered in numerous types of cancer, and the expression pattern was cell specific. Down‐regulation of miR‐181b had been detected in lung cancer and prostate cancer, while up‐regulation of miR‐181b had been demonstrated in acute myeloid leucemia and oral carcinoma.^21^, ^22^, ^23^, ^24^ Previous studies conducted in patients with colon cancer reported that miR‐181b was overexpressed in tumour samples compared with the normal samples,^25^, ^26^, ^27^, ^28^, ^29^ and especially there was a positive correlation between high expression of miR‐181b and poor survival of patients with stage III colon cancer.^27^, ^30^ The Warburg effect is a well‐known feature in cancer‐specific metabolism, and it has been extensively studied over the past 10 years. Several strategies and small molecules which are capable of inhibiting glycolytic metabolism of cancer cells have promising anticancer activity in vitro and in vivo.^31^, ^32^ In the present study, we investigated the role of miR‐181b in colon cancer on the aspect of the Warburg effect. The in vitro experiments for the first time demonstrated that miR‐181b increased the glucose consumption and lactic acid production, suggesting that miR‐181b promoted the Warburg effect in colon cancer cells. Furthermore, the in vivo experiments revealed that miR‐181b promoted the growth of colon cancer xenografts in mice. These findings were consistent with the previous studies about colon cancer,^25^, ^26^, ^27^, ^29^ which firmly validated the oncomiR role of miR‐181b in colon cancer. The overexpression of miR‐181b promoted colon cancer by regulating target genes and it was related to poor prognosis and therapeutic outcome. However, in gastric cancer and prostatic cancer, miR‐181b was found to suppress tumour via inhibiting glycolysis.^12^, ^13^ Therefore, it was also indicated that the role of miR‐181b in different tumours was varied.

PIAS3 is a member of the PIAS family of transcriptional modulators. It is involved in regulating STAT3 signalling via inhibiting the DNA‐binding activity of STAT3.^33^ PIAS3 also functions as a small ubiquitin‐like modifier (SUMO)‐E3 ligase which catalyses the covalent attachment of a SUMO protein to specific target substrates. PIAS3 physically interacts with pyruvate kinase M2 (PKM2),^34^ one of the rate‐controlling glycolytic enzymes that catalyse the transfer of a phosphoryl group from phosphoenolpyruvate (PEP) to adenosine diphosphate (ADP), generating pyruvate and adenosine triphosphate (ATP). These studies suggest that PIAS3 may be involved in the regulation of glycolysis. In the present study, we found PIAS3 overexpression decreased the r glucose consumption and lactic acid production in vitro, and inhibited the growth of colon cancer xenografts in vivo. Furthermore, the promotive effect of miR‐181b on the Warburg effect and tumour growth could be reversed by PIAS3 knockdown. These findings indicated the role of PIAS3 in the Warburg effect, and demonstrated that miR‐181b promoted the Warburg effect and the growth of colon cancer xenografts by targeting PIAS3.

STAT3 is frequently activated in human tumours. Upon stimulation, STAT3 becomes phosphorylated by the receptor associated kinases on a unique tyrosine, and then forms homo‐ or heterodimers that are translocated to the nucleus where they regulate the transcription of specific target genes, such as cyclin D1, B‐cell lymphoma‐2 (Bcl‐2), B‐cell lymphoma‐extra large (Bcl‐xL), matrix metalloprotein‐2 (MMP2), and vascular endothelial growth factor (VEGF).^35^ Increased levels of phosphorylated STAT3 have been detected in the majority of human tumours and tumour cell lines. It has been well‐documented that the aberrant activity of STAT3 can promote cell survival, angiogenesis, immune escape, and metastasis.^36^ PIAS3 protein is a specific inhibitor of STAT3. In the present study, we validated that miR‐181b activated STAT3 in colon cancer cells, and furthermore PIAS3 mediated the effect of miR‐181b on STAT3 activation. These results thus indicated miR‐181b/PIAS3/STAT3 as a novel axis in maintaining the Warburg effect in colon cancer carcinogenesis.

**Figure 7:**
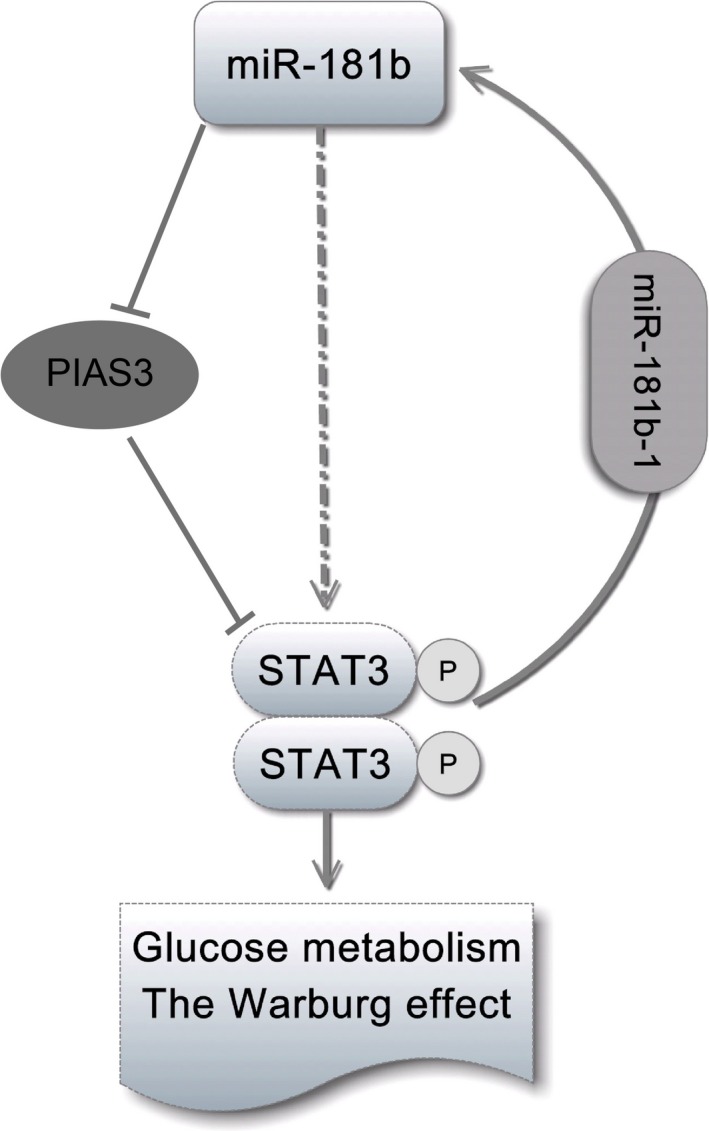
A scheme shows the positive feedback loop between miR‐181b and STAT3 regulating the Warburg effect in colon cancer

Importantly, this study provided a new evidence on the role of miR‐181b‐mediated positive feedback loops in STAT3 activation. We demonstrated that miR‐181b directly bound to the PIAS3 3′UTR and inhibited the expression of PIAS3, and PIAS3 suppression contributed to the activation of STAT3. The proline, isoleucine, asparagine, isoleucine, threonine (PINIT) motif is one of several conserved domains of the PIAS family.^37^, ^38^ The PINIT domain allows nuclear translocation of PIAS3^37^, ^38^ and it is known to promote STAT3‐PIAS3 interaction.^39^ A short stretch of 50 amino acids in the PINIT domain of PIAS3 (PIAS3_82‐132_) can directly interact with STAT3 and down‐regulate STAT3‐dependent transcriptional activity.^37^, ^38^ The (His)(7) ‐PINIT domain (PIAS3_85‐272_) alone is shown to specifically bind to STAT3 in a concentration‐dependent manner.^39^ L97 and R99 are part of a potential binding surface and mutations of L97 and R99 of the PINIT domain abrogate binding to STAT3.^39^ In addition, the acidic region of the carboxyl terminal of PIAS3 can specifically bind to the rPP‐C8 region of STAT3, thereby inhibiting the proliferation and promoting the apoptosis of tumour cells.^40^ Therefore, miR‐181b might inhibit the expression of PIAS3 and directly bind to the PINIT domain of PIAS3 (PIAS3_82‐132_, PIAS3_85‐272_, and/or L97&R99). Then the PIAS3 suppression consequently reduced the binding of PIAS3 to functional domain of STAT3 such as rPP‐C8. Eventually, the phosphorylation of STAT3 was enhanced. Previous publications reported that STAT3 directly activated the transcription of miR‐181b‐1, a precursor of miR‐181b, leading to the increased expression of miR‐181b.^19^, ^41^, ^42^ Based on these findings, we proposed and explored the hypothesis that miR‐181b expression interacted with STAT3 phosphorylation in a positive feedback loop that affected the aerobic glycolysis in colon cancer cells via regulating PIAS3 expression (Figure 7). We investigated the relationship between miR‐181b, PIAS3, and STAT3 expression, and the results from the overexpression and knockdown experiments provided support for our hypothesis. This hypothesis not only clarified the mechanism underlying the role of miR‐181b in the activation of STAT3, but also provided a theoretical basis for the persistent activation of STAT3 in colon cancer.

Based on our animal experiment results in vivo, simple intraperitoneal injection of the various constructs can indeed affect tumours derived from cells which were not previously (i.e. in vitro) transiently transfected, but it is necessary to further study whether it can be applied to clinical treatment (or whether intraperitoneal injection is equally effective for clinical treatment). A previous publication revealed that JAK/STAT3 inhibitors could down‐regulate the miR‐181b expression in oesophageal cancer cells and it was inducible by cytokines activating STAT3.^42^ In colon cancer cells, to date, there is no clear answer to whether JAK can affect miR‐181b expression and whether it is induced by STAT3 activator, which needs to be further studied. We will explore this issue in next study.

In conclusion, this study for the first time demonstrated that miR‐181b contributed to the Warburg effect and xenograft tumour growth of colon cancer by targeting PIAS3. Moreover, a positive feedback loop between miR‐181b and STAT3 regulating the Warburg effect in colon cancer was demonstrated. These findings highlighted the role of miR‐181b/PIAS3/STAT3 axis in tumorigenesis, and provided novel approaches for colon cancer treatment.

## CONFLICT OF INTEREST

The authors confirm that there is no conflict of interest.

## AUTHORS’ CONTRIBUTIONS

XLP, JF, and ZHZ did the most experiments and contributed to the writing of manuscript. LHY and SJM conducted qPCR and Western blot experiments. BH prepared the figures and performed the statistical analysis. XLP, JF, ZHZ, and GXZ planned the project and participated in coordination. All authors read and approved the final manuscript.
