# Supplementary material for: A positive feedback loop between miR‐181b and STAT3 that affects Warburg effect in colon cancer via regulating PIAS3 expression
Source: J Cell Mol Med. 2018 Jul 28;22(10):5040–9. doi: 10.1111/jcmm.13786 (PMC6156246; doi:10.1111/jcmm.13786)
Supplement: Supplementary file 1 [file JCMM-22-5040-s001.docx]

Supporting information for

**A positive feedback loop between miR-181b and STAT3 that affects Warburg effect in colon cancer via regulating PIAS3 expression**

**Xiaolin Pan^a#^, Jin Feng^b#^, Zhenhua Zhu^a#^, Linhua Yao^c^, Shijie Ma^d^, Bo Hao^d^ and Guoxin Zhang^d*^**

^a^Department of Gastroenterology, the First Affiliated Hospital of Nanchang University, Nanchang 330006, Jiangxi, China

^b^Department of General Surgery, the Third Affiliated Hospital of Soochow University, Changzhou 213003, Jiangsu, China

^c^Department of Gastroenterology, the First Affiliated Hospital of Huzhou Teachers College, Huzhou 313000, Zhejiang, China

^d^Department of Gastroenterology, the First Affiliated Hospital of Nanjing Medical University, Nanjing 210029, Jiangsu, China

^#^The authors equally contributed to this work

*****Correspondence to: Guoxin Zhang, Department of Gastroenterology, the First Affiliated Hospital of Nanjing Medical University, Nanjing 210029, Jiangsu, China. Phone and Fax: +86-25-6813603-6532.Email: guoxinzhang11@126.com


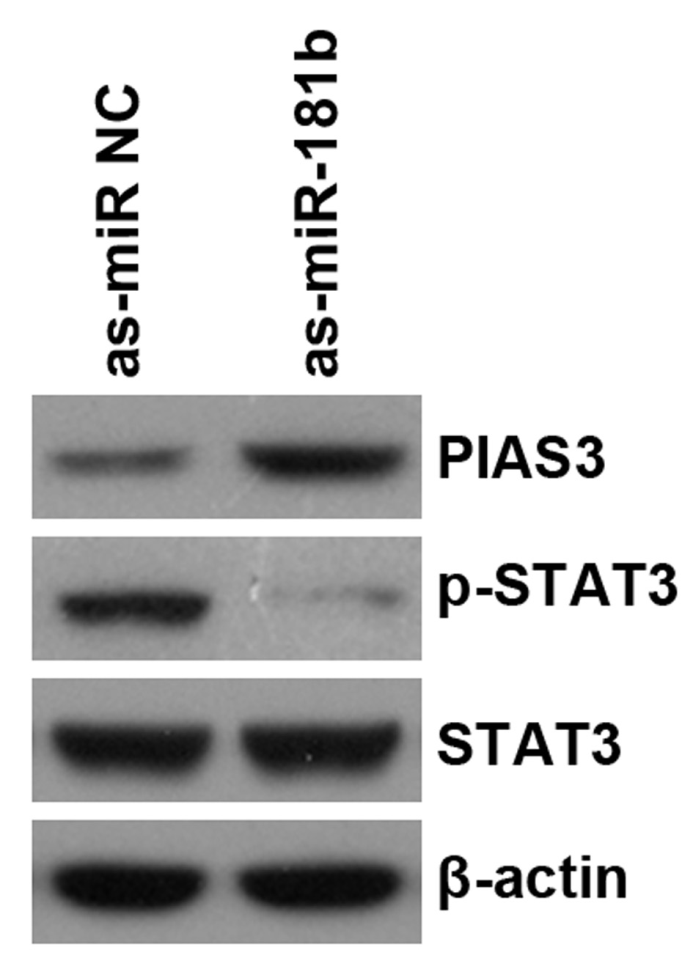


**Fig.S1** Protein expression of PIAS3, STAT3, and phosphorylated STAT3 (p-STAT3) in HT-29 cells following transfection with the miR-181b inhibitor.


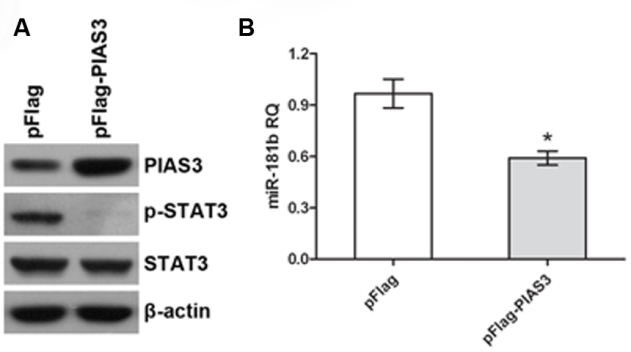


**Fig.S2** Effect of PIAS3 on p-STAT3 and miR-181b expression in HT-29 cells. (A) PIAS3 overexpression suppresses p-STAT3 expression. (B) PIAS3 overexpression suppresses miR-181b expression. *P <0.05. RQ: relative quantity.


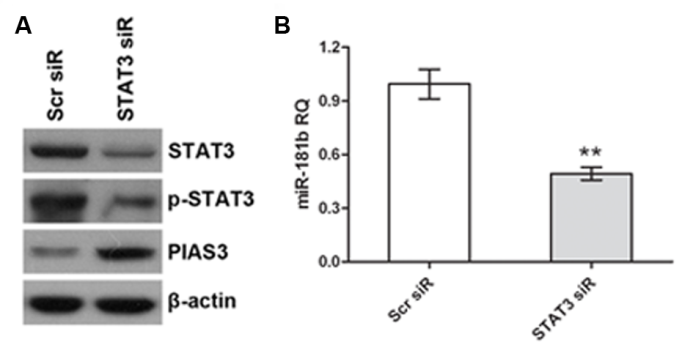


**Fig.S3** Effect of STAT3 on miR-181b and PIAS3 expression in HT-29 cells. (A) STAT3 suppression upregulates PIAS3. (B) STAT3 suppression downregulates miR-181b. ***P* <0.01. RQ: relative quantity.
